# Prediction of Mechanical Properties for Carbon fiber/PLA Composite Lattice Structures Using Mathematical and ANFIS Models

**DOI:** 10.3390/polym15071720

**Published:** 2023-03-30

**Authors:** Mustafa Saleh, Saqib Anwar, Abdulrahman M Al-Ahmari, Abdullah Yahia AlFaify

**Affiliations:** Industrial Engineering Department, College of Engineering, King Saud University, P.O. Box 800, Riyadh 11421, Saudi Arabia; msaleh3@ksu.edu.sa (M.S.); alahmari@ksu.edu.sa (A.M.A.-A.); aalfaify@ksu.edu.sa (A.Y.A.)

**Keywords:** carbon fiber-reinforced PLA, composites, biodegradable polymer, additive manufacturing, FDM, TPMS lattice structures, compression testing, specific energy absorption, artificial intelligence, ANFIS

## Abstract

This study investigates the influence of design, relative density (RD), and carbon fiber (CF) incorporation parameters on mechanical characteristics, including compressive modulus (E), strength, and specific energy absorption (SEA) of triply periodic minimum surface (TPMS) lattice structures. The TPMS lattices were 3D-printed by fused filament fabrication (FFF) using polylactic acid (PLA) and carbon fiber-reinforced PLA(CFRPLA). The mechanical properties of the TPMS lattice structures were evaluated under uniaxial compression testing based on the design of experiments (DOE) approach, namely, full factorial design. Prediction modeling was conducted and compared using mathematical and intelligent modeling, namely, adaptive neuro-fuzzy inference systems (ANFIS). ANFIS modeling allowed the 3D printing imperfections (e.g., RD variations) to be taken into account by considering the actual RDs instead of the designed ones, as in the case of mathematical modeling. In this regard, this was the first time the ANFIS modeling utilized the actual RDs. The desirability approach was applied for multi-objective optimization. The mechanical properties were found to be significantly influenced by cell type, cell size, CF incorporation, and RD, as well as their combination. The findings demonstrated a variation in the E (0.144 GPa to 0.549 GPa), compressive strength (4.583 MPa to 15.768 MPa), and SEA (3.759 J/g to 15.591 J/g) due to the effect of the studied variables. The ANFIS models outperformed mathematical models in predicting all mechanical characteristics, including E, strength, and SEA. For instance, the maximum absolute percent deviation was 7.61% for ANFIS prediction, while it was 21.11% for mathematical prediction. The accuracy of mathematical predictions is highly influenced by the degree of RD deviation: a higher deviation in RD indicates a lower accuracy of predictions. The findings of this study provide a prior prediction of the mechanical behavior of PLA and CFRPLA TPMS structures, as well as a better understanding of their potential and limitations.

## 1. Introduction

Lattice structures are attracting attention in a wide range of industries, including aerospace, automotive, medical, and heat management, where lightweight and multifunctionality are required. Properties like thermal insulation, acoustic absorption, mechanical vibration damping, high stiffness-to-volume fraction ratio, and energy absorption are required within these structures [1]. They enable the enhancement of the performance-to-weight ratio, the creation of highly controlled architectures, and the distribution of impact shock across an object.

In comparison to bulk materials, lattice structures have a high number of design variables, which increases their complexity and limits their ability to be fabricated using conventional manufacturing processes. However, the advent of additive manufacturing (AM) provides opportunities to fabricate such complex structures, e.g., lattice structures, and opens doors for creating and exploring more designs. 

The fundamental advantage of the lattice structures is that materials can be placed only where they are needed for a particular application [2]. Thus, the inherent characteristics of the material, relative density (RD), and geometry variables are the most important factors that influence various lattices’ performances, all of which can be tuned to satisfy the needed qualities. In other words, material, RD, and geometry are variables that can be varied to achieve specific lattice properties. In regards to material, AM allows for controlling material composition, e.g., incorporating particles/fibers as reinforcement, leading to uncovering new enhanced multiphase (composite) materials. This enhances the functionality and performance of the lattices in many aspects, such as mechanical [3,4,5,6], medical [6,7], electrical [8], and multifunctional characteristics [9]. In this regard, the FDM technique offers promising potential for developing highly reliable and mechanically strong composite lattice structures [10]. 

The most common lattice structures’ unit cells are strut-based and surface-based, e.g., triply periodic minimal surfaces (TPMS) [11,12]. Strut-based structures are characterized by their structural members’ joint and frame architecture [13]. Examples of strut-based cell types are body-centered cubic (BCC), face-centered cubic (FCC) [2], octet-truss [14], octagonal, and Kelvin [15]. On the other hand, surface-based structures have a sheet-like architecture. Examples of TPMS cell topologies are Diamond, Gyroid, Primitive, Fisher-Koch, and IWP [16]. Compared with strut-based structures, TPMS structures have shown better mechanical performance, such as high strength, high load-bearing capacity, high energy absorption capacity, and structural stability [17]. Furthermore, TPMS structures have a higher surface-to-volume ratio, enabling them to be used in medical fields (e.g., scaffolds [18,19]) and heat management [20]. 

Researchers are becoming increasingly interested in the design and 3D printing of TPMS structures in an attempt to enhance their performance. Spear and Palazotto [13] investigated the impact of different parameters including cell topologies (Diamond, Primitive, and I-WP), size and number of cells, and wall thickness on the mechanical performance of TPMS structures using factorial design. The samples were printed by selective laser melting (SLM) using INC718 material. The results indicated the importance of considering the main and combined variables when designing a lattice structure, especially for energy absorbing purposes. Kladovasilakis et al. [21] studied the mechanical behavior of three TPMS structures, namely Gyroid, Diamond, and Primitive, printed with PLA by FDM. The different structures were investigated at different RDs (10% to 30%). They found that Diamond structures showed the highest strength, whereas Primitive structures demonstrated the highest capacity for energy absorption. At low RDs, the mechanical properties of the considered TPMS structures deteriorate. Abueidda et al. [22] studied the effect of TPMS cell topologies (Primitive, Schoen IWP, and Neovius), structure size, and RD on the stiffness and strength of SLS-made TPMS polyamide-12 under compression testing. Results showed that both Neovius and IWP structures demonstrated improved strength and stiffness compared with Primitive structures. Shi et al. [23] researched the compression properties and energy absorption of four TPMS cell topologies (Gyroid, Diamond, IW, and Primitive). The TPMS structures were additively manufactured at different RDs from Ti6Al4V using SLM. Compression strength and modulus, and energy absorption were influenced by cell topologies and RDs, according to the findings. Ali et al. [24] investigated the effects of annealing heat treatment and various cell topologies, including Diamond, Primitive, Gyroid, Split-P, Kelvin, Octet, and Sea-Urchin Plus (SUP), on surface morphology, mechanical characteristics, and energy absorption. The results showed that among the studied structures, the SUP structure demonstrated the highest strength, while the Diamond lattice demonstrated the best energy absorption capacity.

Regarding the incorporation of reinforcements as a strategy for enhancing lattices’ performance, very limited work has been reported particularly in TPMS structures. Zarei Zarei et al. [6] explored the effect of adding Ti6Al4V to PLA on the mechanical and biological performance of FDM-printed scaffolds. The results demonstrated that the ultimate compressive strength and compressive modulus of composite scaffolds were enhanced by the incorporation of 3–6 wt% Ti6Al4V. Qin et al. [17] studied the influence of adding reinforcements (CaCO3 and TCP) to the PLA matrix on the compressive modulus, compressive strength, energy absorption, structure stability of Diamond (TPMS type) and cubic structures, additively manufactured by FDM. Results showed that the strength and compressive modulus of lattice structures were enhanced by adding CaCO3 and TCP. Furthermore, with regard to cell topology, the Diamond lattice demonstrated better load-bearing capacity, energy absorption, and structure stability. Kaur et al. [3] performed compression testing to study the mechanical performance of octahedral and octet lattice structures printed with PLA and carbon fiber-reinforced composite of PLA (CFRPLA) using FDM. CFRPLA’s octahedral and octet structures demonstrated higher modulus and energy absorption than PLA structures. Based on their observation, this could be due to shear forces acting on the polymer melt during extrusion causing fibers to align along the printing direction, enhancing the structures’ mechanical stability. Stan et al. [10] investigated the mechanical behavior of FDM 3D-printed lattice structures under axial and transverse compression tests. Structures were 3D-printed from carbon fiber (CF) and glass fiber (GF) reinforced polyamide-12 (PA12) composites. Considering axial and transverse specific load and stiffness, the performance of the CF/PA12 structures was better than that of the PA12 or GF/PA12 structures. In [25], experiments and numerical simulations were used to study the bending properties of various lattice structures made of wood fiber/PLA. Hexagonal, squared, triangular, circular-cored hexagonal, and circular-core squared structures were considered. The results showed that the circular-cored hexagonal structure yielded the highest flexural strength and stiffness. However, this study did not compare the findings of wood fiber/PLA with neat PLA to show the influence of wood fiber incorporation.

It is critical to understand the single and combined effects of various variables, such as material characteristics, relative density, and design parameters on the performance of the FDM TPMS structures. Studying the single and combined effects of various variables needs to be conducted based on a design of experiments (DOE) scheme. This allows statistical exploration of the single and combined effects of factors and further generalization of the lattice’s performance using model-based analysis. Developing models based on factorial design could be a beneficial method for designing customized lattice structures [26]. To the best of our knowledge, almost all reported studies of TPMS lattice structures have not employed a DOE to investigate the influence of design, materials, and relative density variables, which have the most impact on the lattice structure’s properties. Only a few studies have used a DOE approach to investigate the performance of strut-based lattice structures [26,27,28,29,30,31] and focused only on single-phase materials. In addition, no study has reported using artificial intelligence modeling to predict the mechanical performance of 3D-printed lattice structures made from singular-phase or composite materials. 

It is worth mentioning that most of the reported studies ignored the influence of RD when statistically investigating the influence of the design parameters on mechanical characteristics. For instance, the reports that studied the effect of design parameters, such as strut/wall thickness, cell type, and cell size directly induced RD variations in their results. In other words, it can be said that the results were affected by RD in an uncontrolled manner. This is because wall thickness, cell type, and cell size control the RD, and any combination of them will lead to a particular RD. For example, [26] performed a statistical analysis of the effect of cell size, cell type, and strut diameter on different mechanical characteristics. Similarly, the authors of [13] conducted a statistical study on the influence of different TPMS topologies, cell sizes, cell numbers, and surface thicknesses. In such cases, the statistical findings could be misleading since the influence may be attributed to the resulting RD rather than the investigated factors. 

This study attempts to examine the influence of material composition, geometry (cell type and size), and RD variables on the FDM-printed TPMS lattice structures using the DOE approach. In this regard, three TPMS cell types, namely Diamond, Gyroid, and Primitive, with various cell sizes (8 mm and 12 mm), CF incorporation (0% and 15%), and RDs (30% and 44%) were considered. The analysis was conducted based on a full factorial design. The actual relative densities of the printed samples were measured to ensure no significant difference between the designed and printed RDs. Uniaxial compression testing was used to evaluate the mechanical properties of 3D-printed samples, including compression modulus, strength, and SEA. Moreover, ANFIS modeling was also employed for predicting the performance of the TPMS structures, and the results were compared with the mathematical models developed based on the DOE approach. By using the ANFIS modeling predictions, we could accurately predict mechanical characteristics considering the inherent imperfections in 3D printing, such as an RD variation, which cannot be avoided. Due to the freedom in the ANFIS modeling approach, the actual RDs were used instead of the intended (designed) RDs, leading to improved prediction accuracy. Finally, the best parameter settings for maximizing the TPMS lattices’ performance were determined using multi-objective optimization through the desirability function. 

The following section discusses the materials and methods used in this study. The results are presented in Section 3. Section 4 provides the discussions. Finally, Section 5 presents the conclusions.

## 2. Materials and Methods

### 2.1. Materials

In this study, the TPMS structures were FDM-fabricated from two materials: (1) pure Polylactic acid (PLA) and (2) carbon fiber-reinforced PLA (CFRPLA) in which 15% high-modulus short carbon fibers CF were incorporated into the PLA matrix. The matrix of both materials is Natureworks 4043D PLA biopolymer grade. The materials (filaments) were provided by 3DXTech, USA, with a diameter of 1.75 mm, and used as received. For the pure PLA filament, the mechanical properties are 1.24 g/cm^3^ (density, ρ), 56 MPa (Tensile strength at break), and 2865 MPa (Tensile Modulus) [32], while for the CFRPLA filament: 1.29 g/cm^3^ (density, ρ), 48 MPa (Tensile strength at break), 4950 MPa (Tensile Modulus) [33].

### 2.2. Design and Relative Density

**Figure 1:**
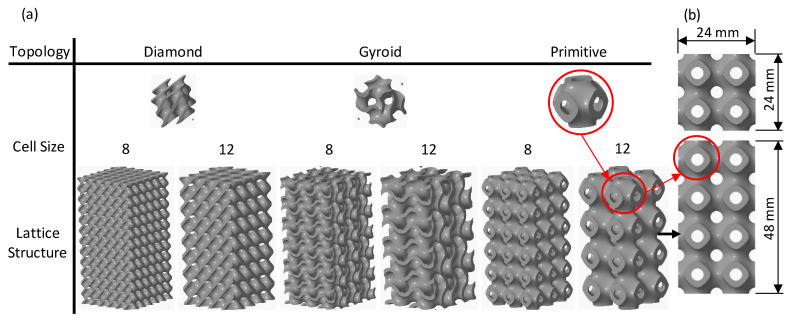
TPMS Lattice structures at 30% RD: (**a**) cell topologies, cell sizes, and the associated lattice structures and (**b**) lattice structure dimensions.

In the current research, three different TPMS cell topologies, including Gyroid (G), Diamond (D), and Primitive (P) were considered. Within the context of this study, the terms “cell topology” and “cell type” are used interchangeably. Figure 1a illustrates the G, D, and P cell topologies. The unit cell size (*l*) of all cell topologies was designed in a cubic unit cell with 8 mm and 12 mm edge lengths. The reason behind these particular lengths is to fit the whole lattice dimensions. 

Relative density (RD) is one of the most factors that influence a lattice’s performance. RD, also termed as volume fraction (V_lattice_/V_overall_), is defined as the lattice structure volume (V_lattice_) divided by the overall structure volume (V_overall_) [34]. This study employed 30% and 44% RDs, taking into account the minimum feasible wall thickness to meet FDM resolution, nozzle diameter, and cell sizes. The selected range of the RDs also considered that the actual densities of the printed samples showed a close agreement with the designed RDs. For each TPMS cell topology, the needed RD is determined by the wall thickness and length of the cell. Hence, the intended RD was controlled by adjusting the ratio of the cell wall thickness parameter (*t*) to the cell size (*t*/*l*) [16]. It should be mentioned that every cell topology has its function in relation to the *t*/*l* ratio because of the differences in surface areas. The t values were determined based on the CAD predictions. Table 1 presents the values of the *t* parameter for each cell topology at the designed RD and cell lengths.

The lattice structures were designed and printed in sizes of 24 × 24 × 48 mm^3^, see Figure 1b. The whole lattice dimensions were selected based on the aspect ratio (length to width ratio) of the ASTM D695-15 standard for compressive testing of rigid polymeric materials (), so that the length should be twice the sample width [17]. The number of cells to fit the selected dimensions depends on the cell size, whether 8 mm (3 × 3 × 6 cells) or 12 mm (2 × 2 × 4 cells). CREO 8.0 software was used to design the G, D, and P structures in the STL format. High-accuracy STL files were obtained for all investigated designs, with the number of generated triangles varying from 464,860 to 6,653,676 depending on the cell topology, cell size, and RD.

### 2.3. Experimental Design

The influence of the material composition (CF incorporation), cell topology, cell size, and relative density on the mechanical properties, including compression modulus (E) and strength, and specific energy absorption (SEA), was evaluated by the DOE approach. Table 2 illustrates the four parameters and their respective levels. A full factorial design was used with 24 runs. Each run was repeated 3 times resulting in a total of 72 experiments. Analysis of variance (ANOVA) was performed with a 95% confidence interval to study the significant influence of the variables and their interactions on E, compressive strength, and SEA. *p*-Values less than 0.05 imply that model terms (main factors and interactions) are statistically significant. Mathematical relationships between the investigated parameters and each of the output responses were developed. The developed mathematical models were further considered for optimization of the considered parameters. The best settings of the considered parameters were optimized through the desirability approach to simultaneously (multi-objective optimization) achieve the maximum E, compressive strength, and SEA. Design-Expert 13 software was used to systematically analyze the influence of the investigated parameters, develop mathematical (prediction) models, and conduct multi-objective optimization.

### 2.4. FDM Printing of TPMS Lattices

**Figure 2:**
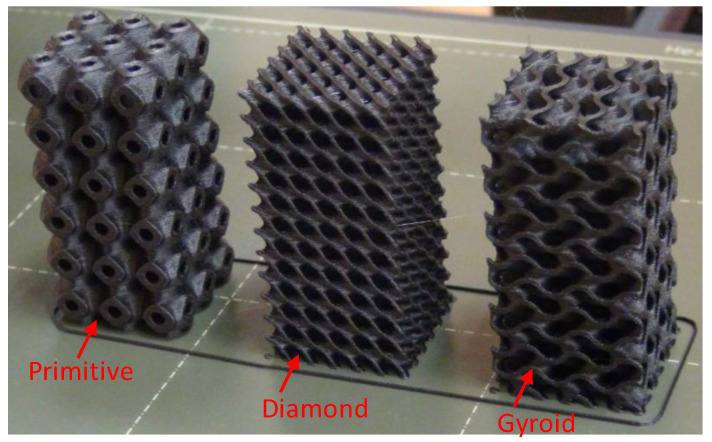
Examples of printed TPMS structures of PLA at 44% RD of different cell topologies.

An open-source FDM machine, Prusa FDM printer (Original Prusa i3 MK3S+, Czech Republic), equipped with a 0.4 mm nozzle, was used to additively manufacture the lattice structures. The STL files for the TPMS lattice structures were imported into slicer software (PrusaSlicer 2.4.2) in order to slice and generate “GCODE” files. Table 3 presents the printing parameters employed to 3D print the samples. All samples were printed without support structures. Examples of printed TPMS structures of different cell topologies are displayed in Figure 2.

### 2.5. Metrological Characterization

**Figure 3:**
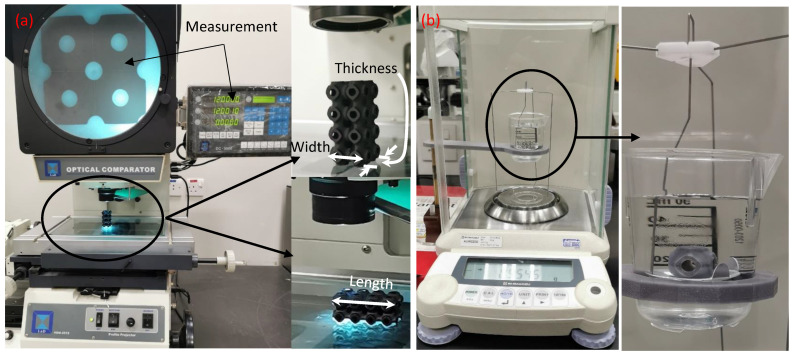
Measuring setups: (**a**) dimension measurements and (**b**) density measurements.

The printed samples’ actual dimensions (e.g., length, width, and thickness) were determined by a Lab Profile Projector (VOM-2515), as shown in Figure 3a. Three measurements were made for each dimension, and the averaged values were used. Density was obtained by utilizing Archimedes’ method, a common method used for determining the density of both porous and solid structures. First, the sample’ density was determined by Archimedes’ method by weighing the sample in air and distilled water. The volume of the lattice (V_lattice_) was then determined from the obtained density. Then, the ratio between (V_lattice_/V_overall_), which represent the RD, was calculated. The overall volume (V_overall_) was calculated from the actual sample’ dimensions. Measurements were conducted on Shimadzu Analytical Balance (AUW220D, China) with a readability of 0.01 mg and a universal specific gravity kit (SGK-C, Mineralab, UK) as in [5]; see Figure 3b. 

### 2.6. Mechanical Properties

The influence of the considered parameters on the mechanical characteristics of the TPMS structures was conducted under a uniaxial compression test. The compression tests were carried out following the ASTM D695-15 standard. Compression tests were performed along the build direction at a constant crosshead speed of 1.6 mm/min up to 60% strain on a Zwick Z100 testing machine equipped with a 100 KN load cell. During the tests, forces and displacements were recorded using testXpert II software. 

Mechanical characteristics, including compressive modulus (E), compressive strength, and SEA, were gathered from the force-displacement curves. First, engineering compressive stress was used and calculated by dividing the recorded forces (F) by the measured original cross-sectional area. The strain was computed by dividing the recorded displacements (δ) during the tests by the original length of the sample. Then, compression modulus and strength were determined. Compression modulus was calculated by the slope of the tangent line at the linear portion of the stress-strain curves by testXpert II software. Compression strength (σ_peak_) was considered as the peak strength, the maximum stress-value of the first peak (i.e., first local maximum) in the stress-strain curve [17,35]. 

Specific energy absorption (SEA) is a useful indicator for measuring a structure’s capability to absorb energy per unit weight. The SEA of a lattice structure is represented by the area under the force-displacement curve divided by the structure’s weight. The SEA was calculated as in Equation (1) [14]. The area under the force-displacement curve was calculated up to 55% strain (theoretical strain densification strain) using MATLAB R2022a.

(1) $$SEA=\frac{\int_{0}^{\delta }Fd\delta }{m}$$

where *F* is the force, *δ* is the displacement, and *m* is the structure’s weight.

### 2.7. Adaptive Neuro-Fuzzy Inference System (ANFIS) Model

**Figure 4:**
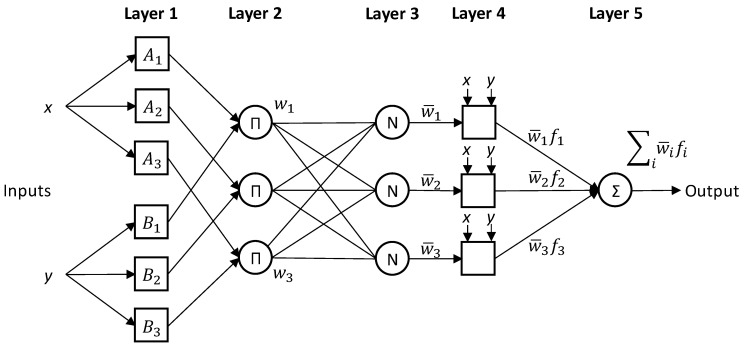
ANFIS structure.

ANFIS is a hybrid neuro-fuzzy method for modeling complex systems. It integrates the best learning abilities of the artificial neural network (ANN) and inference capabilities of the fuzzy inference system (FIS) [36,37]. ANFIS accomplishes sample-based learning using the train data set to develop an efficient ANFIS structure for solving the associated problem. The developed ANFIS structure is being evaluated for its validity through a test data set. ANFIS uses a five-layer, feed-forward propagation structure [38]. Figure 4 shows an illustration of the ANFIS structure with two inputs, three membership functions (MFs), and one output. The layer explanation is described as follows [38,39,40]:

Layer 1: input membership functions (MFs). In this layer, also called the fuzzification layer, the fuzzy membership value of each input, i.e., $\mu A_{i}(x)$ and $\mu B_{i}(y)$ is calculated by a proper membership function, e.g., trapezoidal, Gaussian, and triangular. 

Layer 2: fuzzy rules. The firing strength ($w_{i})$, the weight for each rule’s output is calculated in this layer. Each node’s output is the product of all its input signals, which can be calculated using Equation (2).

(2) $$w_{i}=\mu A_{i}\left(x\right)\times \mu B_{i}\left(y\right),i=1,2,3$$

Layer 3: normalization. This layer represents the normalization of the firing strength ($\bar{w_{\iota }}$), as computed by Equation (3).

(3) $$\bar{w_{\iota }}=\frac{w_{i}}{\sum_{i}w_{i}},i=1,2,3$$

Layer 4: defuzzification. The output of each node in this layer is calculated based on the function given in Equation (4).

(4) $$\bar{w_{\iota }}\cdot f_{i}=\bar{w_{\iota }}(p_{i}x+q_{i}y+r_{i}),i=1,2,3$$

where $p_{i}$, $q_{i}$, and $r_{i}$ called a consequent parameter set.

Layer 5: output layer. This layer has only one node to calculate the system output, as in Equation (5).

(5) $$Output=\sum_{i}\bar{w_{\iota }}\cdot f_{i}$$

**Figure 5:**
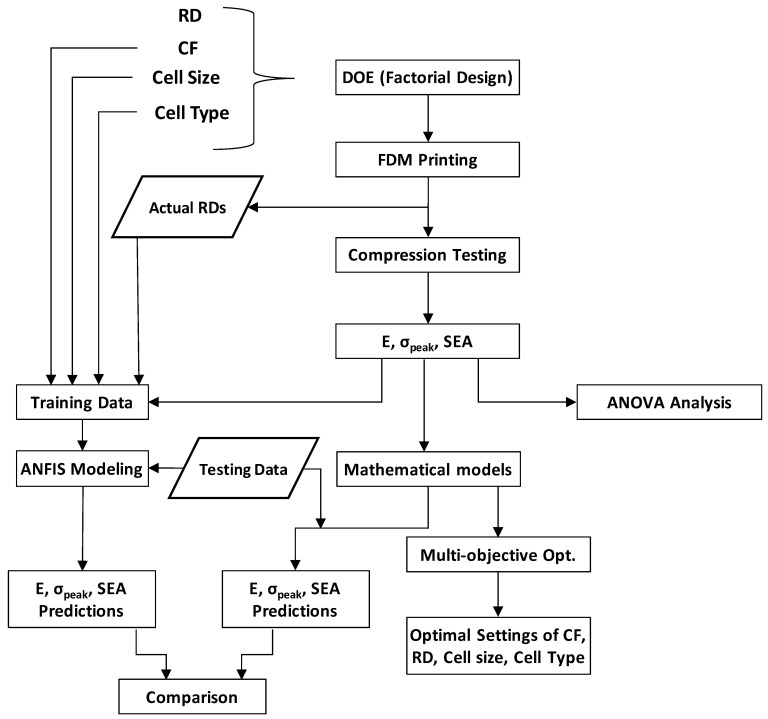
Methodology adopted in this study.

The ANFIS modeling was used for predicting the performance of the TPMS structures in terms of E, σpeak, and SEA. Using the ANFIS modeling predictions, we could accurately predict mechanical characteristics considering imperfections in 3D printing, such as an RD variation. This way, the actual RDs were used instead of the intended RDs, leading to improved prediction accuracy. Figure 5 depicts the methodology adopted in this study.

## 3. Results

### 3.1. Mechanical Characterization

**Figure 6:**
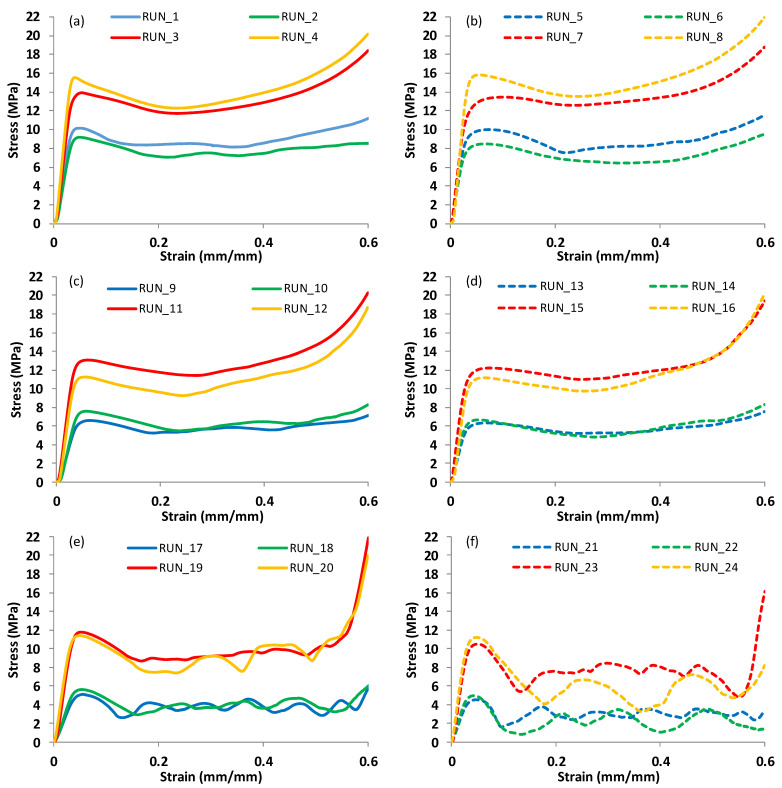
Typical stress-strain curves of the experimental runs listed in Table 4, classified based on the cell type and CF %: (**a**,**b**) Diamond-based samples at 0% CF and 15% CF, respectively; (**c**,**d**) Gyroid-based samples at 0% CF and 15% CF, respectively; (**e**,**f**) Primitive-based samples at 0% CF and 15% CF, respectively.

Table 4 shows the average values of the actual RDs, E, σ_peak_, and SEA of the 24 runs according to the full factorial DOE. The variabilty in the three repeated measured results is indicated by the standard deviation (SD). The results, including E, σ_peak_, and SEA, were derived from the force-displacement curves. Typical stress-strain curves under the uniaxial compression test of the 24 runs listed in Table 4 are depicted in Figure 6. As can be seen in Figure 6, the mechanical behavior of the lattice structures is susceptible to changes in the CF incorporation, design, and RD factors. This influence is shown by how much the stress-strain curves vary in terms of either the stress range or the shape of the curves. For instance, the influence of the cell type in terms of the stress range and the deformation behavior is shown between Diamond-based structures (Figure 6a), Gyroid-based structures (Figure 6c), and Primitive-based structures (Figure 6e). For example, Diamond-based structures show higher load-bearing capacity and more uniformity in deformation in comparison with Primitive-based structures.

**Figure 7:**
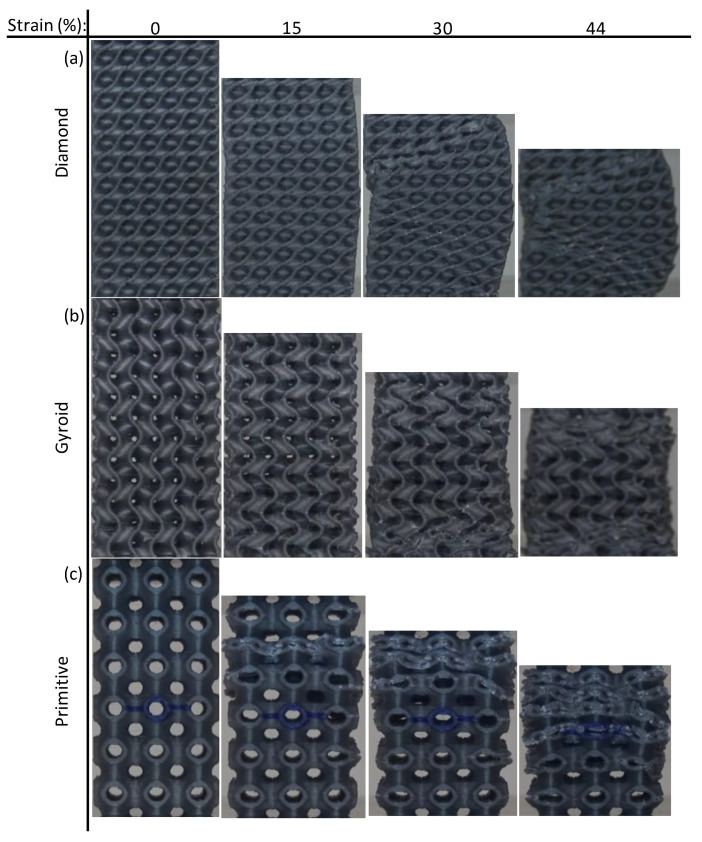
Failure mechanisms of different structures at different strain % of samples with 30% RD, 8 mm cell size, and 0% CF: (**a**) Diamond, (**b**) Gyroid, and (**c**) Primitive.

Deformation mechanisms are graphically illustrated in Figure 7. A bending–torsion coupled failure is evident for the Diamond-based structures; see Figure 7a. Figure 7b shows that the Gyroid-based structure exhibits bending and buckling mechanisms of failure. Figure 7c illustrates the deformation of the Primitive-based structure showing a layer-by-layer deformation mechanism as also reflected by the stress-strain curves; see Figure 6e,f.

### 3.2. ANOVA Analysis

**Figure 8:**
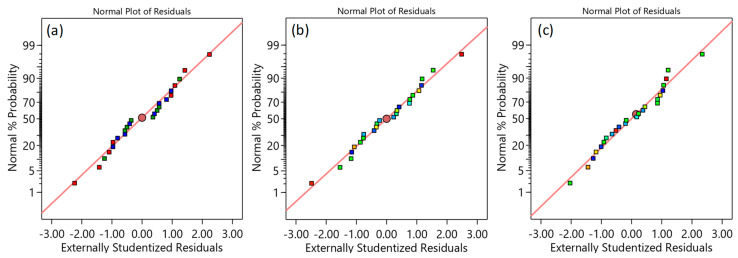
Normality plot of (**a**) E, (**b**) σ_peak_, and (**c**) SEA.

**Figure 9:**
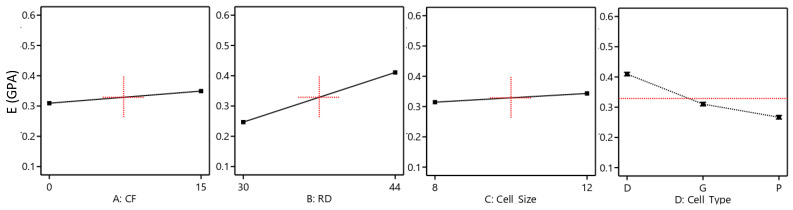
Effect of the main factors on the E.

**Figure 10:**
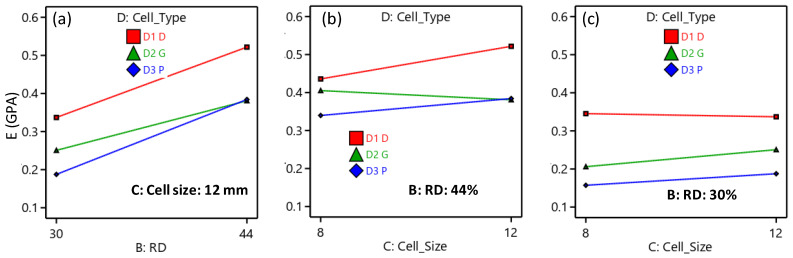
Interaction plots: (**a**) BD interaction, (**b**) CD interaction, and (**b**,**c**) BCD interaction.

The effect of the considered variables on the TPMS structure performances was studied statistically using ANOVA. Reduced ANOVA tables were utilized so that nonsignificant terms were eliminated using backward/forward methods to enhance the model accuracy without sacrificing the model fit. It is worth mentioning that the normality assumption was satisfied (see Figure 8). The $R^{2}$ of E, σ_peak_, and SEA are 0.995, 0.999, and 0.97, respectively, which indicates an excellent representation of the variability of the data by the model terms. Table 5 shows the reduced ANOVA table of the compressive modulus, and *p*-values less than 0.05 indicate that model terms are significant. From the ANOVA table (Table 5), all considered factors, including CF incorporation, RD, cell type, and cell size, significantly influence the E. Two-source interactions, including the cell type and RD, and cell type and cell size, show a significant influence on the E. Furthermore, three-source interactions, namely RD, cell size, and cell type, have a significant influence on the E. The most significant effects are caused by changing the RD variable (58.95%), followed by cell type (31.32), and then the CF incorporation (3.51%). Figure 9 and Figure 10 also provide a visual representation of the results, showing the influence direction of the main factors (Figure 9) and their interactions (Figure 10) on the compressive modulus. The Diamond cell type demonstrates the highest compressive modulus, followed by the Gyroid cell type, and finally, the Primitive cell type, which demonstrated the lowest compressive modulus performance. The other parameters—CF, RD, and cell size—have a proportional influence, meaning that a change from a low level to a high level of any of them can increase in the E. Figure 10b,c show how the interaction of the RD, cell size, and cell type affects the E. For instance, the E of the Diamond-based structures improved at high RD (44%) (Figure 10b) but decreased at low RD (30%) (Figure 10c) as the cell size increased.

**Figure 11:**
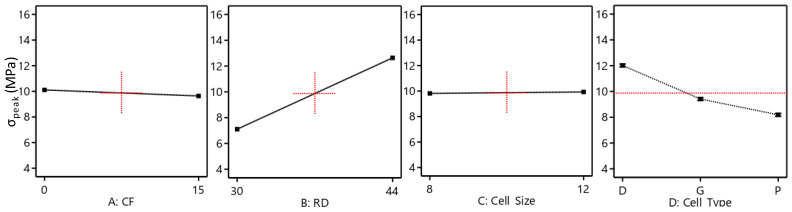
Influence of the main factors on the σ_peak_.

**Figure 12:**
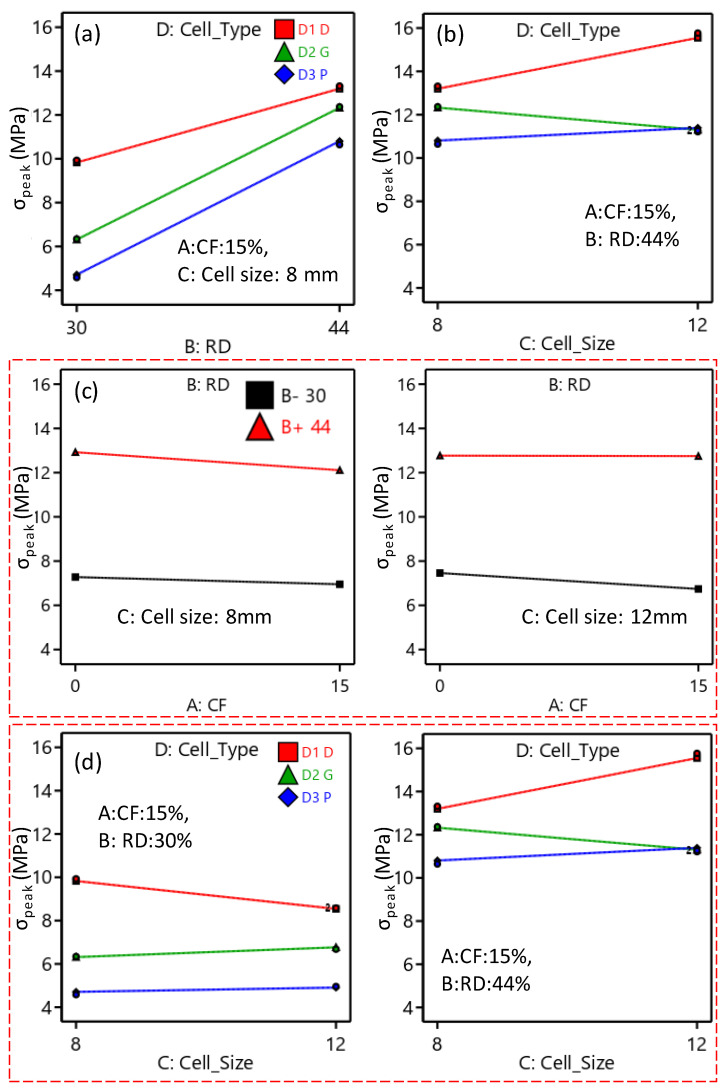
Interaction influence on the σ_peak_: (**a**) BD interaction, (**b**) CD interaction, (**c**) ABC interaction, and (**d**) BCD interaction.

Similarly, the reduced ANOVA table for the σ_peak_, peak strength is presented in Table 6. According to the σ_peak_ ANOVA table, the factors that significantly influence σ_peak_ are CF incorporation, RD, and cell type. The two-source interaction terms, including RD and cell type, and cell size and cell type, have a significant influence on the σ_peak_. Furthermore, RD and cell size interact significantly with the other two parameters, CF and cell type. The most significant effects are caused by changing the RD variable (71.68%), followed by cell type (23.87%). Figure 11 demonstrates the directional influence of the main components, whereas Figure 12 shows the two- and three-source interaction terms. Figure 11 shows that the TPMS structures with Diamond-cell type exhibit the highest σ_peak_, whereas the Primitive-based cell type structures show the lowest. Compressive strength is proportionally influenced by RD; increasing RD results in an increase in σ_peak_ (Figure 11). Regarding CF, Figure 11 demonstrates that σ_peak_ decreases as CF increases. The interaction between cell type and size is illustrated in Figure 12b, which demonstrates that as cell size increases, the σ_peak_ of the Diamond and Primitive structures improve while the σ_peak_ of the Gyroid structures decreases. Figure 12d shows how the interaction of the RD, cell size, and cell type affects the σ_peak_. For instance, the σ_peak_ of the Diamond-based structures improved at high RD (44%) but decreased at low RD (30%) as the cell size increased.

**Figure 13:**
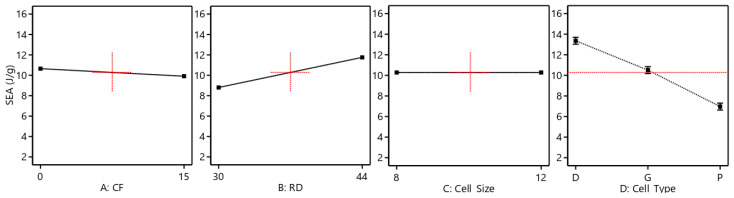
Influence of the main factors on the SEA.

**Figure 14:**
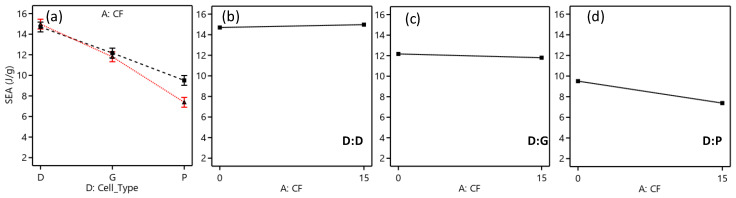
Varying impact of the CF % on the SEA based on cell type at a cell size of 12 mm and RD of 44%: (**a**) CF and cell type interaction, (**b**) Diamond cell type, (**c**) Gyroid Primitive cell type, and (**d**) Primitive cell type.

The reduced ANOVA table for SEA is presented in Table 7. The results indicate that the cell type, CF incorporation, and RD have a significant influence on the SEA. In addition, the two-source interaction between the cell type and CF has a significant influence on the SEA. The most important effects are contributed by the cell type (70.51%), followed by RD (22.44%), and then the CF and cell type interaction (2.66%). The influence of the main factors is also graphically presented in Figure 13. Similar to the compressive modulus and strength, Diamond-based cell types exhibit the best SEA. SEA is proportionally influenced by RD, i.e., an increase in RD results in an upsurge in SEA. Figure 13 demonstrates that SEA decreases as CF increases. This influence, however, varies depending on the cell type, as illustrated in Figure 14. For instance, Diamond-based TPMS structures show an enhancement in SEA with an increase in CF (Figure 14a,b), whereas Primitive-based TPMS structures show a decrease in SEA (Figure 14a,d). Furthermore, Table 7 demonstrates the significant influence of the interaction between the CF and cell type (shown in Figure 14a) on SEA, providing further evidence that CF’s influence on SEA is dependent on the cell topology. 

### 3.3. Mathematical and ANFIS Prediction Models

Mathematical relationships between the responses, including E, σ_peak_, and SEA, and the investigated variables were developed based on the reduced models in the ANOVA analysis, e.g., Table 5, Table 6 and Table 7. The developed mathematical models are presented in Table 8.

Similarly, the ANFIS models were developed to predict the performance of the TPMS structures in terms of E, σ_peak_, and SEA. An advantage of this type of modeling is that it eliminates the requirement to strictly adhere to predetermined settings for any variable in the DOE, where any change in that setting will most likely impact the results. For instance, the ANOVA analysis results (Section 3.2) proved that RD contributed the most to the mechanical properties, including E, σ_peak_, and SEA. Thus, a variation in the actual RD from the designed RD will certainly lead to a variation in the mechanical properties. In this regard, actual RD should be used instead of using the designed one, particularly when there is a high variation, which cannot be avoided. Using the actual RDs makes the analysis and modeling more accurate. Variations between the actual and designed RDs are attributed to a number of reasons depending on the AM process. For instance, deviation in wall thickness and the presence of voids and cracks are reasons to deviate RD in the FDM process [5,41]. Excess porosity is an example of the source RD deviation in the SLS process, as elaborated by [42]. In laser-powder bed fusion (L-PBF), unmelted powder particles that have no pathway to get out of the structure, closed pores due to powder clog [43], deviation in wall thickness, voids and cracks, and surface roughness [16] are reasons for RD deviation. Therefore, a modeling approach such as ANFIS will be more accurate as it can consider the measured RDs instead of the designed ones.

In this study, the ANFIS models were developed during the training phase based on the full factorial results (as training data) presented in Table 4. The ANFIS technique allows for the utilization of input data even if it does not comply with a specific DOE. It should be noted that the actual RDs were used instead of the designed RDs when developing ANFIS models in both the training and testing phases. Thus, more realistic models could be developed, resulting in improved prediction accuracy. The accuracy of the developed models was evaluated with the testing data set presented in Table 9. For each TPMS performance (e.g., E, σ_peak_, and SEA), the accuracy of the developed ANFIS models was evaluated using the root mean square error (RMSE), which was calculated using Equation (6).

(6) $$RMSE=\sqrt{\frac{1}{n}\sum_{i=1}^{n}{\left({Exp}_{i}-{Pred}_{i}\right)}^{2}}$$

where n is the number of testing data while ${Exp}_{i}$ and ${Pred}_{i}$ are the experimental and predicted results of the ith testing experiment, respectively. 

**Figure 15:**
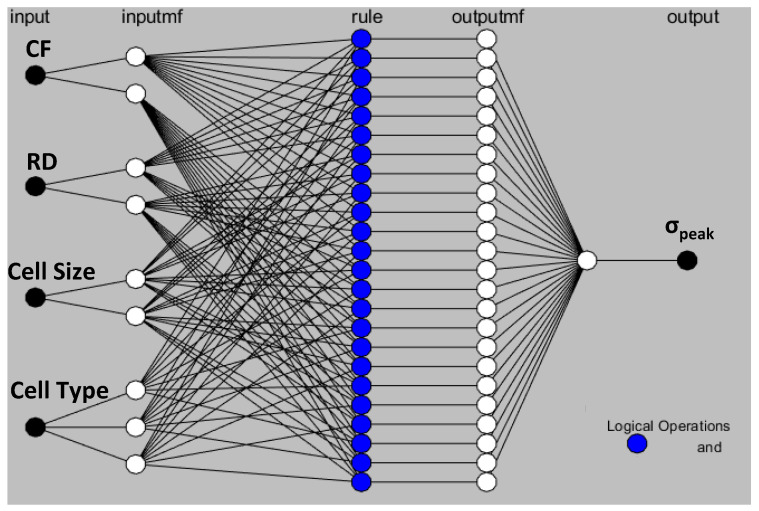
ANFIS structure network for modeling the σ_peak_ with “2 2 2 3” number of MFs.

Furthermore, for each TPMS performance, RMSE was used during the training phase for tuning and selecting the fuzzy inference parameters to minimize the RMSE. The fuzzy inference parameters and their settings that resulted in the lowest RMSE are presented in Table 10. An illustration of the ANFIS structure of the σ_peak_ response is provided in Figure 15. 

**Figure 16:**
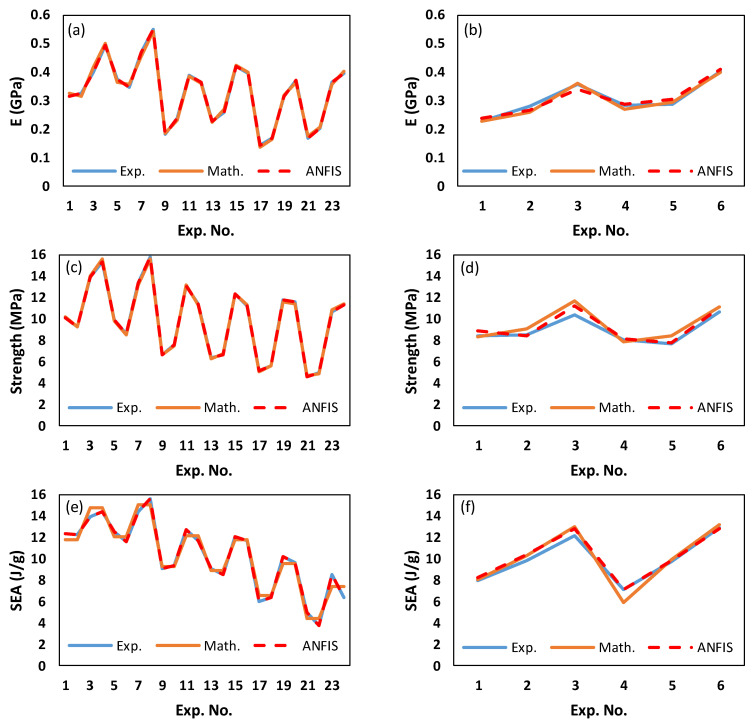
Comparison between experimental, mathematical, and ANFIS results for the training and testing data sets: (**a**) training data of E, (**b**) testing data of E, (**c**) training data of σ_peak_, (**d**) testing data of σ_peak_, (**e**) testing data of SEA, and (**f**) training data of SEA.

**Figure 17:**
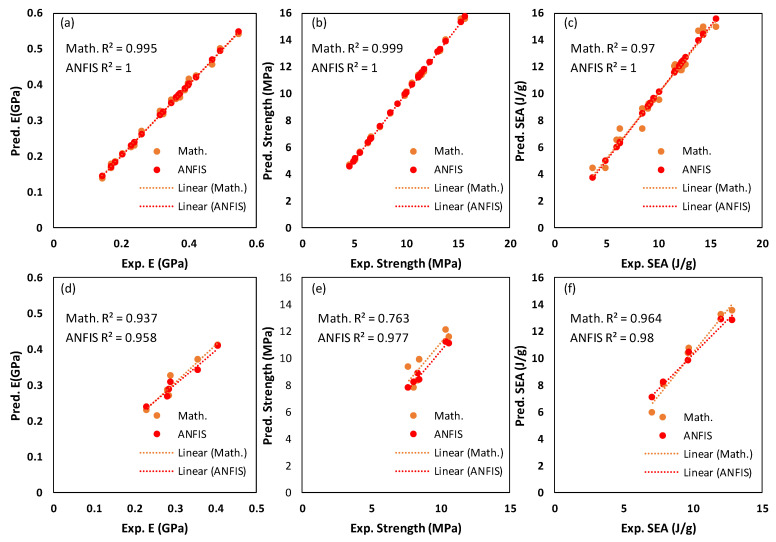
Experimental results vs. mathematical and ANFIS predicted results for the training and testing data sets: (**a**) training data of E, (**b**) training data of σ_peak_, (**c**) training data of SEA, (**d**) testing data of E, (**e**) testing data of σ_peak_, and (**f**) testing data of SEA.

In Figure 16, the predicted values for E, σ_peak_, and SEA based on the mathematical and ANFIS models are shown alongside the experimental results. Figure 16a,c,e depict the experimental and predicted findings for the training data, demonstrating that experimental and predicted results obtained from both methods are comparable. Figure 17a–c show evidence of the close agreement between predicted results obtained by both models and the experimental results in terms of $R^{2}$ for E, σ_peak_, and SEA. Similarly, Figure 16b,d,f depict the experimental and predicted findings for the testing data, showing that the predicted results obtained from ANFIS methods are much close to the experimental results. Furthermore, an enhancement is clearly shown in ANFIS predicting results for E, σ_peak_, and SEA compared with mathematical results. For instance, the $R^{2}$ of ANFIS prediction for σ_peak_ is 0.977, which is significantly higher than that of mathematical prediction, which is 0.763. 

Table 11 compares the RMSE performance of the mathematical and ANFIS models with respect to testing experiments, revealing that ANFIS models have outperformed mathematical models in all responses. Furthermore, a comparison between mathematical and ANFIS results for each test experiment in terms of absolute percent deviation ($Dev.=\left|\frac{{Exp}_{i}-{Pred}_{i}}{{Exp}_{i}}\right|*100$) is presented in Table 12. From Table 12, the maximum deviation in the ANFIS’ prediction results was 7.61% (the 3rd test experiment, σ_peak_), while a deviation of 21.11% (the 5th test experiment, σ_peak_) was found for the mathematical model. It should be noted that the maximum deviation in the mathematical prediction results (21.11%) occurred in the 5th testing experiment, which showed a high RD deviation (actual and designed RDs are 37% and 34.91%, respectively). Furthermore, mathematical models also displayed some deviations exceeding 10%, contrary to ANFIS deviation values. 

The results shown in Figure 16 and Figure 17 and Table 11 and Table 12 highlight the significance of employing AI models such as the ANFIS model for predicting the performances of TPMS lattice structures while accounting for the issues associated with 3D printing, such as RD deviation. Table 12 shows that whenever the actual RD is close to the designed RD, both models provide accurate predictions. However, for a high variation in RD, the ANFIS model is preferable. This indicates the ability of both modeling approaches to predict various mechanical properties of TPMS structures while considering different variables, including material composition, geometry, and RD.

### 3.4. Multi-Objective Optimization

Desirability analysis was used to select the best settings of the CF incorporation, relative density, cell type, and cell size RD that led to maximizing the σ_peak_, E, and SEA. Table 13 shows the optimal combination values of the considered variables for multi-objective optimization. Diamond topology, 12 mm cell size, 15% CF, and 44% RD should be used to achieve an overall desirability of 97.8%. Nevertheless, if, for whatever reason, either Gyroid or Primitive cell topologies are selected, the optimal combinations as well as the overall desirability values for both designs are illustrated in Table 14. Table 13 and Table 14 present the significance of carefully selecting the cell topology, cell size, and CF incorporation, as different combinations of these factors result in varying performances. 

Table 15 shows the validation experiments related to the multi-objective optimization findings reported in Table 13 and Table 14, demonstrating a good agreement between the multi-objective optimization and experimental results. 

## 4. Discussion

Stress-strain curves depicted in Figure 6 show evident variations in the mechanical response during the compression testing. Variations are clearly detected in terms of stress, compressive modulus, and deformation patterns. Primitive structures showed a wave pattern of deformation (Figure 6e,f (e.g., run #17–24)), while Diamond structures (Figure 6a,b) and Gyroid structures (Figure 6c,d) seemed to deform uniformly, giving them the advantage of accumulating the load-bearing capacity (e.g., run #1–8 for Diamond and run #9–16 for Gyroid. Compared with D (Figure 6a) and G (Figure 6c), samples with 0% CF, which exhibit a sharp reduction in stress following elastic deformation, D (Figure 6b) and G (Figure 6d) samples with 15% CF demonstrate more plastic deformation. However, Figure 6f shows that incorporating CF into the Primitive structures, particularly with samples of 12 mm cell sizes (run#22 and run#22 24), makes the deformation more wavy than in samples with 0% CF (Figure 6e).

**Figure 18:**
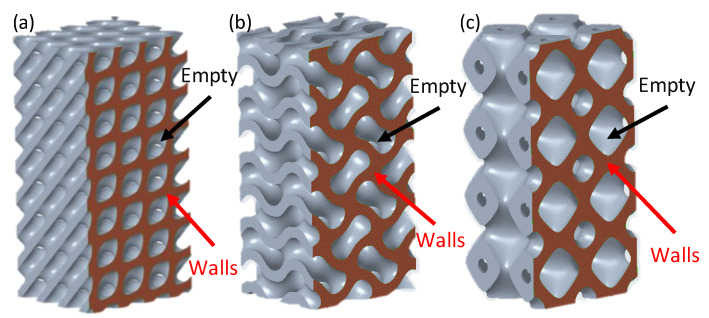
Cross-sectional of Lattice structures showing material distribution and empty spaces at 12 mm cell size and 44% RD: (**a**) Diamond, (**b**) Gyroid, and (**c**) Primitive.

From the results presented in Table 4, Diamond-based TPMS lattice structures showed the best mechanical properties, including compressive modulus, σ_peak_, and SEA. For instance, the maximum compressive modulus (0.549 GPa), σ_peak_ (15.768 MPa), and SEA (15.591 J/g) were observed with Diamond, 15% CF, 44% RD, and 12 mm cell size (run #8). On the other hand, the worst mechanical responses were obtained by Primitive-based cell-type TPMS structures. The minimum compressive modulus (0.144 GPa) was obtained with Primitive, 0% CF, 30% RD, and 8 mm cell size (run #17), while the lowest σ_peak_ (4.583 MPa) was observed with Primitive, 15% CF, 30% RD, and 8 mm cell size (run #21). Similarly, the minimum SEA (3.759 J/g) was observed with Primitive, 15% CF, 30% RD, and 12 mm cell size (run #22). The walls’ orientation and better material distribution within the geometry of the Diamond structures improve wall contact and reduce the empty spaces, making it less susceptible to fracture initiation [5,24]. For illustration, Figure 18 shows cross-sections of lattice structures with 12 mm cell size and 44% RD. It is evident from the cross sections that empty spaces between walls in the Diamond structure (Figure 18a) are less than those in Gyroid and Primitive structures; Figure 18b,c, respectively.

The influence of all considered parameters, including cell topology and size, CF incorporation, and RD, as well as their different combinations on the compressive response of the TPMS structures, was statistically significant. Results showed that RD had the greatest impact on mechanical response among the four studied variables, followed by cell type and CF incorporation. Cell size had the least impact. The authors of [44] stated that RD is the key factor in mechanical performance, including elastic modulus and strength of a given lattice structure. This is in line with the statistical findings, which show that RD has a high influence on both the E and σ_peak_ (58.95% and 71.68%, respectively). The statistical analysis confirms that cell type and RD influence the compressive modulus, σ_peak_, and SEA of the TPMS lattice structures. This finding is in line with previous studies that investigated the effect of TPMS cell topologies and RDs on E, σ_peak_, and SEA characteristics, such that these properties were enhanced as RD increased [16]. Diamond structures showed the best performance, while Primitive structures were the worst [24]. In this regard, when statistically investigating the influence of design parameters, such as strut/wall thickness and cell type and size, RD has to be controlled; otherwise, the results could be misleading.

**Figure 19:**
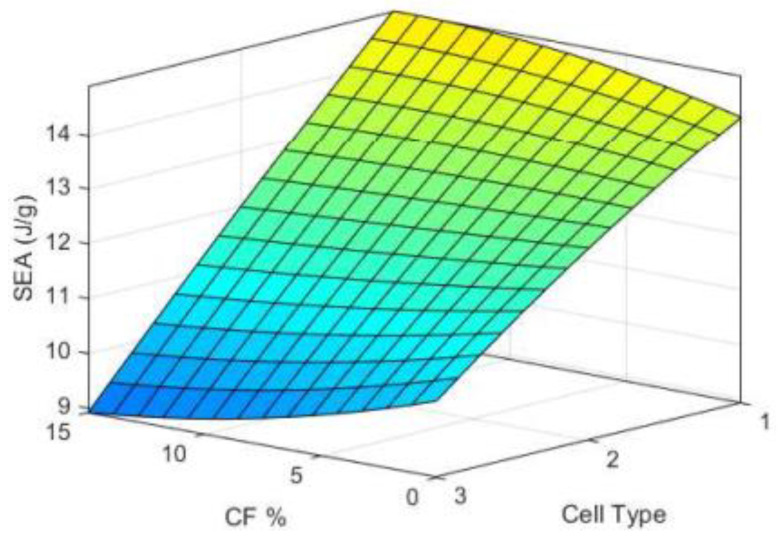
ANFIS 3D surface plot of SEA variation with the CF and cell type interaction at a cell size of 12 mm and RD of 44%; 1, 2, and 3 denote Diamond, Gyroid, and Primitive, respectively.

CF incorporation was found to be a statistically significant influence on the mechanical properties and SEA. These findings are consistent with [3]: CF-reinforced PLA lattices showed enhanced compressive modulus and energy absorption. According to results in [3], the shear forces acting on the polymer melt during extrusion cause fibers to align along the printing direction, enhancing the structures’ mechanical stability. Furthermore, results reported in [4] stated that the tensile modulus and energy absorption at the break of chiral structures were significantly enhanced (by two times) when incorporating CF into PLA. The interaction of CF incorporation with the cell topology significantly influenced the SEA. This finding agrees with [5] in that the CF incorporation evidently enhanced the energy absorption in the octahedral lattices, while a slight influence was found on the octet lattices. This study confirms these previous findings by demonstrating that CF increases the SEA of Diamond-based structures while (Figure 14b) decreasing the SEA of Primitive-based structures (Figure 14d). This influence of CF and cell type interaction on the SEA is also depicted in Figure 19 using ANFIS 3D surface plot.

Cell size had the smallest effect, and the statistical analysis confirmed its influence only on the compressive modulus. However, its interactions with cell type and RD significantly influenced the compressive modulus and strength, as seen in Table 5 and Table 6. Moreover, the influence of the combination of cell size, CF incorporation, and RD on the σ_peak_ was significant. Even though the combination of the three factors had no statistical influence on the SEA contrary to [28] (Truncated octahedron lattices), the negative influence on SEA was consistent with [28]. Findings in [41] stated that certain combinations of wall thickness and cell size (which control the structure RD) of Gyroid structures outperformed others, suggesting the need for predicting the optimal combination of wall thickness and cell size. This observation is noteworthy since it implies that different combinations of design, RD, and material composition (e.g., CF incorporation) factors could be used to attain the desired performance. 

For the training data set, factorial design experiments presented in Table 4, both prediction models, mathematical and ANFIS models, performed well in predicting E, σ_peak_, and SEA, as illustrated in Figure 16 and Figure 17. However, regarding the testing data set presented in Table 9, ANFIS models clearly outperformed the mathematical models in terms of RMSE (Table 11) and the absolute percent deviation (Table 12). Furthermore, Table 12 and Table 16 demonstrate that mathematical modeling predictions deviated more from experimental results compared with ANFIS predictions, notably for experiments having high RD deviation. This implies a connection between mathematical prediction performance and RD deviation. From the ANOVA analysis, Section 3.1, the RD influenced E, σ_peak_, and SEA with a contribution of 58.95%, 71.68%, and 22.44%, respectively. 

**Figure 20:**
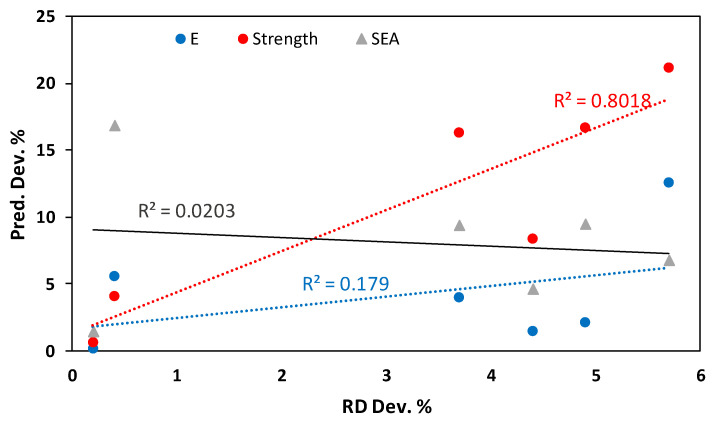
Relationship between RD absolute percent deviation, RD contribution, and mathematical prediction absolute percent deviation.

A correlation was observed between the contribution percentage of the RD influence on the TPMS structure performance (e.g., E, σ_peak_, and SEA) reported in ANOVA analysis (Table 5, Table 6 and Table 7) and the performance of the mathematical predictions. In other words, a high contribution percentage of RD influence on a TPMS structure’s performance (e.g., E, σ_peak_, or SEA) indicates a high error in mathematical modeling predictions for experiments having a high RD deviation. For instance, the higher mathematical modeling prediction deviations for σ_peak_ (16.67%, 16.23%, 21.11%, and 8.30%) were observed for the testing experiments (2, 3, 5, and 6, respectively), with relatively high RD deviations; see Table 16. The fourth testing experiment (i.e., regarding the mathematical prediction deviations for the E and SEA) was an exceptional case where the aforementioned phenomenon was not valid. Furthermore, Figure 20 depicts the relationship between the RD deviation and the mathematical prediction deviation for E, σ_peak_, and SEA. A high correlation between the RD deviation and mathematical prediction deviation in the case of σ_peak_ (R^2^ = 0.802) can be observed, while a low correlation is in the case of SEA. Table 17 presents the results of the Spearman Rho correlation test, indicating a significant correlation (0.943) between the RD deviation and mathematical prediction performance in the case of σ_peak_, which is highly influenced by the RD variable (71.68%). 

## 5. Conclusions

In this study, the influence of different TPMS-based cell topologies with varying unit cell sizes, relative densities, and CF incorporation on the mechanical and specific energy absorption was statistically investigated. The FDM 3D-printed lattices were tested using a uniaxial compression testing, and their E, σ_peak_, and SEA were evaluated. Prediction models were developed using ANFIS and mathematical modeling. By using the ANFIS models, we were able to predict mechanical characteristics considering imperfections in 3D printing, such as an RD variation. This was achieved by using the actual RDs instead of the designed RDs. Multi-objective optimization was conducted using the desirability approach with the objective of maximizing E, σ_peak_, and SEA. The following inferences can be made from this study’s findings:

- The findings demonstrated a change in the E (0.144 GPa to 0.549 GPa), σ_peak_ (4.583 MPa to 15.768 MPa), and SEA (3.759 J/g to 15.591 J/g) due to the impact of the considered variables.
- RD had a significant influence on both E and σ_peak_, with a contribution of 58.95% and 71.68%, respectively. The cell type had the highest impact on the SEA, contributing to 70.51% of the total influence. In general, RD had the highest influence on mechanical responses among the four studied variables, followed by cell type, CF incorporation, and finally, cell size having the least impact.
- The findings revealed the importance of statistically evaluating the influence of the design, RD, and material composition (e.g., CF incorporation) parameters and their combination to attain the desired TPMS lattice structure performance.
- For the training data set (factorial design experiments), both mathematical and ANFIS models predicted E, σ_peak_, and SEA well. However, when it comes to the testing data set (validation experiments), ANFIS models clearly outperformed mathematical models in terms of RMSE and absolute percent deviation in predicting all mechanical characteristics. For instance, the maximum absolute percent deviation was 7.61% for ANFIS prediction, while it was 21.11% for mathematical prediction.
- The accuracy of mathematical predictions is highly influenced by the degree of RD deviation; a higher deviation in RD results in lower accuracy of predictions. Furthermore, a correlation between the mathematical models’ prediction accuracy and the RD deviation was found when the RD influence contribution on a TPMS performance was high. For instance, when RD accounted for 71.68% of the variation in σ_peak_, there was a significant correlation (94.3%) between the accuracy of mathematical predictions and RD deviation. Therefore, for a high variation in RD, ANFIS models are preferable.
- Whenever the actual RD is close to the designed RD, both models provide accurate prediction models. This also indicates the ability of both models to predict different mechanical properties of TPMS structures, taking into account different variables, including material composition, geometry, and the RD.
- This study provides a better understanding of PLA and CFRPLA TPMS structures, as well as an ability to better predict their mechanical behavior. For instance, based on the multi-objective optimization using the desirability approach, the Diamond topology, 12 mm cell size, 15% CF, and 44% RD combination are the best settings that achieved an overall desirability of 97.8% for E, σ_peak_, and SEA.

## Figures and Tables

**Table 1:** Values of wall thickness parameter (*t*) for each TPMS topology at designed RDs and lengths (*l*).

Cell Topology	*l* = 8 mm		*l* = 12 mm	
	RD: 30	RD: 44	RD: 30	RD: 44
D	0.5374	0.7856	0.8065	1.1786
G	0.6826	0.995	1.024	1.493
P	0.772	1.131	1.1585	1.6965

**Table 2:** Materials, design, and RD parameters and their levels.

Parameter	Levels		
	1	2	3
CF incorporation (CF), wt.%	0	15	-
Relative density (RD), %	30	44	-
Cell size, mm	8	12	-
Cell topology (Cell type)	D	G	P

**Table 3:** FDM printing parameters.

Printing Temperature	Bed Temperature	Printing Speed	Layer Thickness	Infill
220 °C	65 °C	45 mm/s	0.2 mm	100%

**Table 4:** Full factorial experiments along with the results (E, σ_peak_, SEA, and actual RDs).

Run#	Variables				Responses			Actual RD (% ± SD)
	A: CF (%)	B: RD (%)	C: Cell Size (mm)	D: Cell Type	E (GPa ± SD)	σ_peak_ (MPa ± SD)	SEA (J/g ± SD)	
1	0	30	8	D	0.315 ± 0.004	10.057 ± 0.204	12.317 ± 0.532	32.36 ± 0.26
2	0	30	12	D	0.325 ± 0.010	9.225 ± 0.054	12.214 ± 0.298	28.50 ± 0.09
3	0	44	8	D	0.402 ± 0.005	13.891 ± 0.027	13.948 ± 0.062	42.65 ± 0.31
4	0	44	12	D	0.495 ± 0.017	15.352 ± 0.173	14.415 ± 0.287	42.58 ± 0.68
5	15	30	8	D	0.375 ± 0.007	9.929 ± 0.149	12.491 ± 0.24	31.18 ± 0.06
6	15	30	12	D	0.349 ± 0.005	8.581 ± 0.166	11.573 ± 0.418	28.11 ± 0.09
7	15	44	8	D	0.470 ± 0.021	13.316 ± 0.027	14.354 ± 0.321	42.35 ± 0.17
8	15	44	12	D	0.549 ± 0.028	15.768 ± 0.199	15.591 ± 0.292	43.33 ± 0.36
9	0	30	8	G	0.182 ± 0.011	6.612 ± 0.129	9.106 ± 0.360	28.91 ± 0.34
10	0	30	12	G	0.240 ± 0.002	7.575 ± 0.034	9.311 ± 0.083	30.48 ± 0.07
11	0	44	8	G	0.389 ± 0.006	13.099 ± 0.082	12.678 ± 0.058	44.99 ± 0.15
12	0	44	12	G	0.364 ± 0.016	11.411 ± 0.196	11.673 ± 0.118	41.68 ± 0.52
13	15	30	8	G	0.230 ± 0.006	6.342 ± 0.111	9.056 ± 0.102	28.51 ± 0.28
14	15	30	12	G	0.262 ± 0.002	6.679 ± 0.045	8.496 ± 0.124	30.18 ± 0.07
15	15	44	8	G	0.421 ± 0.008	12.367 ± 0.206	12.044 ± 0.074	44.15 ± 0.66
16	15	44	12	G	0.398 ± 0.032	11.214 ± 0.108	11.702 ± 0.108	42.55 ± 0.49
17	0	30	8	P	0.144 ± 0.005	5.164 ± 0.074	6.016 ± 0.216	28.50 ± 0.27
18	0	30	12	P	0.171 ± 0.001	5.580 ± 0.090	6.327 ± 0.051	28.59 ± 0.20
19	0	44	8	P	0.315 ± 0.010	11.778 ± 0.096	10.150 ± 0.061	43.48 ± 0.07
20	0	44	12	P	0.37 0 ± 0.006	11.541 ± 0.127	9.636 ± 0.156	42.99 ± 0.58
21	15	30	8	P	0.170 ± 0.004	4.583 ± 0.051	4.985 ± 0.089	28.11 ± 0.13
22	15	30	12	P	0.204 ± 0.005	4.957 ± 0.035	3.759 ± 0.164	28.55 ± 0.07
23	15	44	8	P	0.364 ± 0.008	10.643 ± 0.255	8.499 ± 0.237	42.51 ± 0.33
24	15	44	12	P	0.398 ± 0.006	11.262 ± 0.071	6.375 ± 0.188	43.37 ± 0.19

**Table 5:** Reduced ANOVA table of E.

Source	Sum of Squares	Contribution %	df	Mean Square	F-Value	*p*-Value	
Model	0.2723	99.52	12	0.0227	190.15	<0.0001	significant
A-CF	0.0096	3.51	1	0.0096	80.43	<0.0001	
B-RD	0.1613	58.95	1	0.1613	1351.86	<0.0001	
C-Cell_Size	0.005	1.83	1	0.005	41.73	<0.0001	
D-Cell_Type	0.0857	31.32	2	0.0429	359.23	<0.0001	
BC	0.0003	0.11	1	0.0003	2.17	0.1686	
BD	0.0027	0.99	2	0.0014	11.32	0.0021	
CD	0.001	0.37	2	0.0005	4.37	0.0402	
BCD	0.0067	2.45	2	0.0033	27.88	<0.0001	
Residual	0.0013	0.48	11	0.0001			
Cor Total	0.2736	100	23				

R^2^: 0.995, Adjusted R^2^: 0.99, and Predicted R^2^: 0.977.

**Table 6:** Reduced ANOVA Table of σ_peak_.

Source	Sum of Squares	Contribution %	df	Mean Square	F-Value	*p*-Value	
Model	255.66	99.88	15	17.04	448.56	<0.0001	significant
A-CF	1.33	0.52	1	1.33	34.97	0.0004	
B-RD	183.47	71.68	1	183.47	4828.63	<0.0001	
C-Cell_Size	0.0775	0.03	1	0.0775	2.04	0.191	
D-Cell_Type	61.1	23.87	2	30.55	804.05	<0.0001	
AB	0.0171	0.01	1	0.0171	0.4495	0.5215	
AC	0.0597	0.02	1	0.0597	1.57	0.2454	
BC	0.0994	0.04	1	0.0994	2.62	0.1444	
BD	1.5	0.59	2	0.75	19.74	0.0008	
CD	0.7668	0.3	2	0.3834	10.09	0.0065	
ABC	0.5298	0.21	1	0.5298	13.94	0.0058	
BCD	6.7	2.62	2	3.35	88.2	<0.0001	
Residual	0.304	0.12	8	0.038			
Cor Total	255.96	100	23				

R^2^: 0.999, Adjusted R^2^: 0.997, and Predicted R^2^: 0.989.

**Table 7:** Reduced ANOVA Table of SEA.

Source	Sum of Squares	Contribution %	df	Mean Square	F-Value	*p*-Value	
Model	225.92	97.02	6	37.65	92.36	<0.0001	significant
A-CF	3.28	1.41	1	3.28	8.03	0.0114	
B-RD	52.26	22.44	1	52.26	128.19	<0.0001	
D-Cell_Type	164.18	70.51	2	82.09	201.37	<0.0001	
AD	6.2	2.66	2	3.1	7.61	0.0044	
Residual	6.93	2.98	17	0.4077			
Cor Total	232.85	100	23				

R^2^: 0.97, Adjusted R^2^: 0.96, and Predicted R^2^: 0.941.

**Table 8:** Mathematical prediction models of E, σ_peak_, and SEA.

Response	Topology	Mathematical Model
E	G	−0.625663+0.00266637A+0.0240777B+0.0481099C−0.00123144BC
	D	0.551474+0.00266637A−0.00698681B−0.0525257C+0.00168178BC
	P	−0.253648+0.00266637+0.011005B−0.000032C+0.000253839BC
σ_peak_	G	−20.3991+0.44013A+0.844853B+1.63904C−0.013642AB−0.0490301AC−0.0475763BC+0.00141501ABC
	D	14.1892+0.44013A−0.0749332B−1.53656C−0.013642AB−0.0490301AC+0.0437816BC+0.00141501ABC
	P	−13.6707+0.44013A+0.584075B+0.575688C−0.013642AB−0.0490301AC−0.0142519BC+0.00141501ABC
SEA	G	2.89235−0.0244981A+0.2108B
	D	5.42397+0.0185767A+0.2108B
	P	0.232705−0.141851A+0.2108B

**Table 9:** Testing data set.

Testing Data Set					
Run#	CF (%)	RD (%)	Cell Size (mm)	Cell Type	Act. RD
Test-1	0	37	8	P	37.07
Test-2	0	37	8	G	35.20
Test-3	0	37	8	D	35.62
Test-4	15	37	8	P	37.14
Test-5	15	37	8	G	34.91
Test-6	15	37	8	D	35.36

**Table 10:** Fuzzy inference parameters settings.

Response	Training Opt. Method	MF Type	Output Function	No. MFs	Epochs
E	Hybrid	trimf	Constant	2 2 3 4	210
σ_peak_	Hybrid	pimf	Linear	2 2 2 3	30
SEA	Hybrid	trimf	Linear	2 2 2 2	30

**Table 11:** RMSE of the mathematical and ANFIS prediction models based on the testing data set.

Response	Math. RMSE	ANFIS’ RMSE
E	0.017	0.012
σ_peak_	1.181	0.418
SEA	0.851	0.416

**Table 12:** Experimental and predicted results of the testing data sets.

No. (RD Dev.%)	E (GPa)			σ_peak_ (MPa)			SEA (J/g)			Dev. (E)		Dev. (Str.)		Dev. (SEA)	
	Exp.	Math.	ANFIS	Exp.	Math.	ANFIS	Exp.	Math.	ANFIS	Math.	ANFIS	Math.	ANFIS	Math.	ANFIS
1(0.2)	0.23	0.23	0.24	8.37	8.33	8.84	7.92	8.03	8.19	0.13	3.93	0.56	5.62	1.37	3.39
2(4.9)	0.28	0.29	0.27	8.48	9.89	8.37	9.77	10.69	10.39	2.09	4.91	16.67	1.21	9.46	6.41
3(3.7)	0.36	0.37	0.34	10.40	12.08	11.19	12.10	13.22	12.83	3.91	4.30	16.23	7.61	9.33	6.08
4(0.4)	0.28	0.27	0.29	8.08	7.76	8.18	7.10	5.90	7.06	5.50	1.08	4.04	1.26	16.81	0.50
5(5.7)	0.29	0.33	0.31	7.70	9.32	7.75	9.67	10.32	9.82	12.52	6.14	21.11	0.69	6.76	1.51
6(4.4)	0.40	0.41	0.41	10.63	11.51	11.05	12.91	13.50	12.81	1.38	0.99	8.30	3.96	4.60	0.78

**Table 13:** Optimal parameter settings obtained by multi-objective desirability analysis.

Cell Topology	Cell Size (mm)	CF (%)	RD (%)	E (GPa)	σ_peak_ (MPa)	SEA (J/g)	Desirability %
D	12	15	44	0.542	15.55	14.978	97.0

**Table 14:** Optimal parameter settings considering Gyroid and Primitive cell topologies.

Cell Topology	Cell Size (mm)	CF (%)	RD (%)	E (GPa)	σ_peak_ (MPa)	SEA (J/g)	Desirability %
G	8.178(~8)	15	44	0.424	12.28	11.80	68.6
	8.06 (~8)	0	44	0.385	13.112	12.168	68.6
P	12	0	44	0.364	11.412	9.508	54.4

**Table 15:** Validation experiments for multi-objective optimization.

Cell Topology	Cell Size (mm)	CF (%)	RD (%)	E (GPa)	σ_peak_ (MPa)	SEA (J/g)
D	12	15	44	0.549	15.768	15.591
G	8	15	44	0.421	12.367	12.044
G	12	0	44	0.364	11.411	11.673
P	12	0	44	0.370	11.541	9.636

**Table 16:** Relationship between RD absolute percent deviation, RD contribution, and mathematical prediction absolute percent deviation.

Test No.	RD Dev. (%)	RD Contribution %		
		58.95	71.68	22.44
		E Math. Dev. (%)	σ_peak_ Math. Dev. (%)	SEA Math. Dev. (%)
1	0.2	0.13	0.56	1.37
2	4.9	2.09	16.67	9.46
3	3.7	3.91	16.23	9.33
4	0.4	5.50	4.04	16.81
5	5.7	12.52	21.11	6.76
6	4.4	1.38	8.30	4.60

**Table 17:** Correlation between RD absolute percent deviation and mathematical prediction absolute percent deviation of E, σ_peak_, and SEA.

		Math. Prediction Dev.		
		E	σ_peak_	SEA
RD Dev.	Correlation	0.486	0.943	0.143
	*p*-value	0.329	0.005	0.787

## Data Availability

The data presented in this study are available on request from the corresponding author.

## References

[1] Červinek O., Werner B., Koutný D., Vaverka O., Pantělejev L., Paloušek D. (2021). Computational approaches of quasi-static compression loading of SS316L lattice structures made by selective laser melting. Materials.

[2] Nazir A., Abate K.M., Kumar A., Jeng J.-Y. (2019). A state-of-the-art review on types, design, optimization, and additive manufacturing of cellular structures. Int. J. Adv. Manuf. Technol..

[3] Kaur M., Yun T.G., Han S.M., Thomas E.L., Kim W.S. (2017). 3D printed stretching-dominated micro-trusses. Mater. Des..

[4] Hu C., Dong J., Luo J., Qin Q.-H., Sun G. (2020). 3D printing of chiral carbon fiber reinforced polylactic acid composites with negative Poisson’s ratios. Compos. Part B Eng..

[5] Saleh M., Anwar S., Al-Ahmari A.M., Alfaify A. (2022). Compression performance and failure analysis of 3D-printed carbon fiber/PLA composite TPMS lattice structures. Polymers.

[6] Zarei M., Dargah M.S., Azar M.H., Alizadeh R., Mahdavi F.S., Sayedain S.S., Kaviani A., Asadollahi M., Azami M., Beheshtizadeh N. (2023). Enhanced bone tissue regeneration using a 3D-printed poly(lactic acid)/Ti6Al4V composite scaffold with plasma treatment modification. Sci. Rep..

[7] Belaid H., Nagarajan S., Teyssier C., Barou C., Barés J., Balme S., Garay H., Huon V., Cornu D., Cavaillès V. (2020). Development of new biocompatible 3D printed graphene oxide-based scaffolds. Mater. Sci. Eng. C.

[8] Dong K., Panahi-Sarmad M., Cui Z., Huang X., Xiao X. (2021). Electro-induced shape memory effect of 4D printed auxetic composite using PLA/TPU/CNT filament embedded synergistically with continuous carbon fiber: A theoretical & experimental analysis. Compos. Part B Eng..

[9] Alam F., Varadarajan K.M., Kumar S. (2020). 3D printed polylactic acid nanocomposite scaffolds for tissue engineering applications. Polym. Test..

[10] Stan F., Sandu I.-L., Fetecau C. 3D Printing and mechanical behavior of Anisogrid composite lattice cylindrical structures; American society of mechanical engineers digital collection, September 30 2022. Proceedings of the ASME 2022 17th International Manufacturing Science and Engineering Conference.

[11] Plocher J., Panesar A. (2020). Effect of density and unit cell size grading on the stiffness and energy absorption of short fibre-reinforced functionally graded lattice structures. Addit. Manuf..

[12] Riva L., Ginestra P.S., Ceretti E. (2021). Mechanical characterization and properties of laser-based powder bed–fused lattice structures: A review. Int. J. Adv. Manuf. Technol..

[13] Spear D., Palazotto A. (2021). Investigation and Statistical Modeling of the mechanical properties of additively manufactured lattices. Materials.

[14] Ling C., Cernicchi A., Gilchrist M.D., Cardiff P. (2019). Mechanical behaviour of additively-manufactured polymeric octet-truss lattice structures under quasi-static and dynamic compressive loading. Mater. Des..

[15] Habib F.N., Iovenitti P., Masood S.H., Nikzad M. (2018). Fabrication of polymeric lattice structures for optimum energy absorption using multi jet fusion technology. Mater. Des..

[16] AlMahri S., Santiago R., Lee D.-W., Ramos H., Alabdouli H., Alteneiji M., Guan Z., Cantwell W., Alves M. (2021). Evaluation of the dynamic response of triply periodic minimal surfaces subjected to high strain-rate compression. Addit. Manuf..

[17] Qin D., Sang L., Zhang Z., Lai S., Zhao Y. (2022). Compression performance and deformation behavior of 3D-printed PLA-based lattice structures. Polymers.

[18] Zhang X.Y., Yan X.C., Fang G., Liu M. (2020). Biomechanical Influence of Structural Variation Strategies on Functionally Graded Scaffolds Constructed with Triply Periodic Minimal Surface. Addit. Manuf..

[19] Yoo D.-J. (2014). Advanced porous scaffold design using multi-void triply periodic minimal surface models with high surface area to volume ratios. Int. J. Precis. Eng. Manuf..

[20] Qureshi Z.A., Al-Omari S.A.B., Elnajjar E., Al-Ketan O., Abu Al-Rub R. (2023). Architected lattices embedded with phase change materials for thermal management of high-power electronics: A numerical study. Appl. Therm. Eng..

[21] Kladovasilakis N., Tsongas K., Tzetzis D. (2021). Mechanical and FEA-Assisted characterization of fused filament fabricated triply periodic minimal surface structures. J. Compos. Sci..

[22] Abueidda D.W., Bakir M., Abu Al-Rub R.K., Bergström J.S., Sobh N.A., Jasiuk I. (2017). Mechanical properties of 3D printed polymeric cellular materials with triply periodic minimal surface architectures. Mater. Des..

[23] Shi X., Liao W., Li P., Zhang C., Liu T., Wang C., Wu J. (2020). Comparison of compression performance and energy absorption of lattice structures fabricated by selective laser melting. Adv. Eng. Mater..

[24] Ali M., Sari R.K., Sajjad U., Sultan M., Ali H.M. (2021). Effect of annealing on microstructures and mechanical properties of PA-12 lattice structures proceeded by multi jet fusion technology. Addit. Manuf..

[25] Zarna C., Chinga-Carrasco G., Echtermeyer A.T. (2023). Bending properties and numerical modelling of cellular panels manufactured from wood fibre/PLA biocomposite by 3D printing. Compos. Part A Appl. Sci. Manuf..

[26] Silva R.G., Estay C.S., Pavez G.M., Viñuela J.Z., Torres M.J. (2021). Influence of geometric and manufacturing parameters on the compressive behavior of 3D printed polymer lattice structures. Materials.

[27] Athanker P., Singh A.K. (2021). Elastic and elasto-plastic analysis of Ti6Al4V micro-lattice structures under compressive loads. Math. Mech. Solids.

[28] Rossiter J.D., Johnson A.A., Bingham G.A. (2020). Assessing the design and compressive performance of material extruded lattice structures. 3D Print. Addit. Manuf..

[29] El-Sayed M.A., Essa K., Ghazy M., Hassanin H. (2020). Design optimization of additively manufactured titanium lattice structures for biomedical implants. Int. J. Adv. Manuf. Technol..

[30] De Pasquale G., Luceri F., Riccio M. (2019). Experimental evaluation of selective laser melting process for optimized lattice structures. Proc. Inst. Mech. Eng. Part E J. Process. Mech. Eng..

[31] Ben Ali N., Khlif M., Hammami D., Bradai C. (2021). Optimization of structural parameters on hollow spherical cells manufactured by fused deposition modeling (FDM) using Taguchi method. Cell. Polym..

[32] PLA Filament. https://www.3dxtech.com/product/ecomax-pla/.

[33] CarbonXTM PLA+CF 3D Filament|Carbon Fiber Reinforced. https://www.3dxtech.com/product/carbonx-pla-cf/.

[34] Guo H., Takezawa A., Honda M., Kawamura C., Kitamura M. (2020). Finite element simulation of the compressive response of additively manufactured lattice structures with large diameters. Comput. Mater. Sci..

[35] Mishra A.K., Chavan H., Kumar A. (2021). Effect of material variation on the uniaxial compression behavior of FDM manufactured polymeric TPMS lattice materials. Mater. Today Proc..

[36] Karaboga D., Kaya E. (2019). Adaptive network based fuzzy inference system (ANFIS) training approaches: A comprehensive survey. Artif. Intell. Rev..

[37] Deshwal S., Kumar A., Chhabra D. (2020). Exercising hybrid statistical tools GA-RSM, GA-ANN and GA-ANFIS to optimize FDM process parameters for tensile strength improvement. CIRP J. Manuf. Sci. Technol..

[38] Xia C., Pan Z., Polden J., Li H., Xu Y., Chen S. (2021). Modelling and prediction of surface roughness in wire arc additive manufacturing using machine learning. J. Intell. Manuf..

[39] Buragohain M., Mahanta C. (2008). A novel approach for ANFIS modelling based on full factorial design. Appl. Soft Comput..

[40] Nasr M.M., Anwar S., Al-Samhan A.M., Ghaleb M., Dabwan A. (2020). Milling of graphene reinforced Ti6Al4V nanocomposites: An artificial intelligence based industry 4.0 approach. Materials.

[41] Maconachie T., Tino R., Lozanovski B., Watson M., Jones A., Pandelidi C., Alghamdi A., Almalki A., Downing D., Brandt M. (2020). The compressive behaviour of ABS gyroid lattice structures manufactured by fused deposition modelling. Int. J. Adv. Manuf. Technol..

[42] Graziosi S., Ballo F.M., Libonati F., Senna S. (2022). 3D Printing of bending-dominated soft lattices: Numerical and experimental assessment. Rapid Prototyp. J..

[43] Zhang C., Banerjee A., Hoe A., Tamraparni A., Felts J.R., Shamberger P.J., Elwany A. (2021). Design for laser powder bed additive manufacturing of AlSi12 periodic mesoscale lattice structures. Int. J. Adv. Manuf. Technol..

[44] Zhao J., Liu H., Zhou Y., Chen Y., Gong J. (2021). Effect of relative density on the compressive properties of Ti6Al4V diamond lattice structures with shells. Mech. Adv. Mater. Struct..

